# Annexin A2 Improves the Osteogenic Differentiation of Mesenchymal Stem Cells Exposed to High-Glucose Conditions through Lessening the Senescence

**DOI:** 10.3390/ijms232012521

**Published:** 2022-10-19

**Authors:** Parin Klabklai, Jitrada Phetfong, Rattanawan Tangporncharoen, Chartchalerm Isarankura-Na-Ayudhya, Tulyapruek Tawonsawatruk, Aungkura Supokawej

**Affiliations:** 1 Department of Clinical Microscopy, Faculty of Medical Technology, Mahidol University, Nakhonpathom 73170, Thailand; 2 Department of Clinical Microbiology and Applied Technology, Faculty of Medical Technology, Mahidol University, Nakhonpathom 73170, Thailand; 3 Department of Orthopaedics, Faculty of Medicine, Ramathibodi Hospital, Mahidol University, Bangkok 10400, Thailand

**Keywords:** mesenchymal stem cells, high glucose, annexin A2, osteogenesis, osteoporosis

## Abstract

Osteoporosis is frequently found in chronic diabetic patients, and it results in an increased risk of bone fractures occurring. The underlying mechanism of osteoporosis in diabetic patients is still largely unknown. Annexin A2 (ANXA2), a family of calcium-binding proteins, has been reported to be involved in many biological process including bone remodeling. This study aimed to investigate the role of ANXA2 in mesenchymal stem cells (MSCs) during in vitro osteoinduction under high-glucose concentrations. Osteogenic gene expression, calcium deposition, and cellular senescence were determined. The high-glucose conditions reduced the osteogenic differentiation potential of the MSCs along with the lower expression of ANXA2. Moreover, the high-glucose conditions increased the cellular senescence of the MSCs as determined by senescence-associated β-galactosidase staining and the expression of p16, p21, and p53 genes. The addition of recombinant ANXA2 could recover the glucose-induced deterioration of the osteogenic differentiation of the MSCs and ameliorate the glucose-induced cellular senescence of the MSCs. A Western blot analysis revealed an increase in p53 and phosphorylated p53 (Ser 15), which was decreased by recombinant ANXA2 in MSC osteoblastic differentiation under high-glucose conditions. Our study suggested that the alteration of ANXA2 in high-glucose conditions may be one of the plausible factors in the deterioration of bones in diabetic patients by triggering cellular senescence.

## 1. Introduction

Osteoporosis is a metabolic bone disease that is characterized by an imbalance of the bone remodeling process due to excessive osteoclastic bone resorption or insufficient osteoblastic bone formation, thereby resulting in a decrease in the bone mass [1]. Osteoporosis is a chronic complication that is found in several certain medical conditions and diseases, particularly diabetic mellitus (DM) [2]. Diabetic mellitus is a metabolic disorder that is characterized by the exposure of the body tissues to elevated levels of blood glucose over a prolonged period. The underlying mechanism of bone mass reduction under high-glucose conditions is largely unclear. The plausible cause of the bone defects in DM is increased bone resorption, which was explained by the induction of osteoclast differentiation and migration in a streptozotocin (STZ)-induced DM mouse model [3,4]. Meanwhile, the mesenchymal stem cells (MSCs) that are isolated from the STZ-induced DM mouse model exhibited a reduction in the matrix mineralization and alkaline phosphatase (ALP) expression when they were cultured with a medium containing 25-and-35 mM glucose [5]. Our previous study revealed that high-glucose conditions reduced the cell viability, inhibited the osteogenic differentiation potential, and altered several protein expressions in the human MSCs [6]. Many mechanisms underlying glucose-induced osteogenic dysfunction have been investigated [7,8]. Triggering cellular senescence has been reported to be an important result of one undergoing a high-glucose condition [9]. Nevertheless, the exact mechanism of the effects of high levels of glucose in the bone-forming cells remains to be explored to develop a therapeutic target for the treatment of osteoporosis.

Annexin A2 (ANXA2) is a group of calcium-dependent phospholipid-binding proteins that are expressed in several cell types, including mononuclear cells [10], cancer cells [11], and bone cells [12]. The biological properties of ANXA2 have been reported in fibrinolysis [13] and bone development in both bone formation and bone resorption [14,15]. Annexin A2 was firstly reported as an autocrine/paracrine factor that facilitates the murine osteoclast formation and bone resorption [16]. In addition, ANXA2 showed an indirect effect on osteoclast proliferation via the triggering of the granulocyte-macrophage colony-stimulating factor (GM-CSF) production in cultured human marrow [14]. On the other hand, osteogenic gene expression and ALP staining were reduced in the ANXA2-deficient preosteoblastic cells, thus suggesting the essential role of ANXA2 in bone formation [17]. Recent evidence showed that ANXA2 in accompany with Forkhead box Q1 (FOXQ1) promoted the osteogenic differentiation of the MSCs through Wnt/beta-catenin signaling [18]. Despite the above evidence, the exact mechanism of ANXA2 in the osteogenic differentiation of the MSCs under a high-glucose condition has not been elucidated.

This study aims to explore the impact of high levels of glucose on ANXA2 expression and the MSC osteoblastic differentiation potential in vitro. Furthermore, the role of recombinant ANXA2 in the improvement of osteogenic differentiation under high-glucose conditions has been investigated. We expected that the findings of this study could provide an enlightened understanding of how high levels of glucose influences osteogenesis through an alteration of ANXA2.

## 2. Results

### 2.1. MSC Characterization

**Figure 1:**
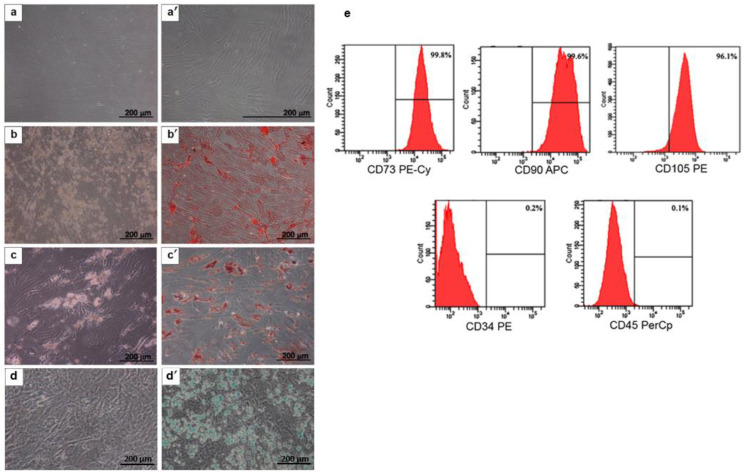
Characterization of human bone marrow mesenchymal stem cells (MSCs). The MSCs show a fibroblast-like morphology (**a**,**a’**). MSCs were cultured in ODM (**b**) and stained with ARS (**b’**). MSCs were cultured in ADM (**c**) and stained with Oil Red O (**c’**). MSCs were cultured in CDM (**d**) and stained with Alcian blue (**d’**). The phenotypic expression of CD34, CD45, CD73, CD90, and CD105 on the MSC surface were evaluated by flow cytometry (**e**).

MSCs that were derived from the bone marrow of healthy donors were characterized based on the minimum criteria that are recommended by the International Society for Cellular Therapy (ISCT) [19], consisting of cell morphology, the mesodermal differentiation potential, and the MSC surface markers. The bone marrow-derived MSCs exhibited plastic adherent properties and showed fibroblast-like morphology in culture flasks after 3 days of being isolated (Figure 1a,a’). For the multilineage differentiation ability, the MSCs were cultured in an osteogenic differentiation medium (ODM), an adipogenic differentiation medium (ADM), and a chondrogenic differentiation medium (CDM) for 10–14 days before they were stained with Alizarin Red S (ARS), Oil Red O, and Alcian blue, respectively. The positive staining for calcium deposition (Figure 1b,b’), fat droplets (Figure 1c,c’), and glycosaminoglycans (Figure 1d,d’) was observed. Immunophenotyping revealed that the MSC was positive for CD73 (99.8%), CD90 (99.6%), and CD105 (96.1%), and it was negative for the hematopoietic lineage markers CD34 (0.2%) and CD45 (0.3%) (Figure 1e).

### 2.2. The Effect of High Levels of Glucose on Osteogenic Differentiation of MSCs

**Figure 2:**
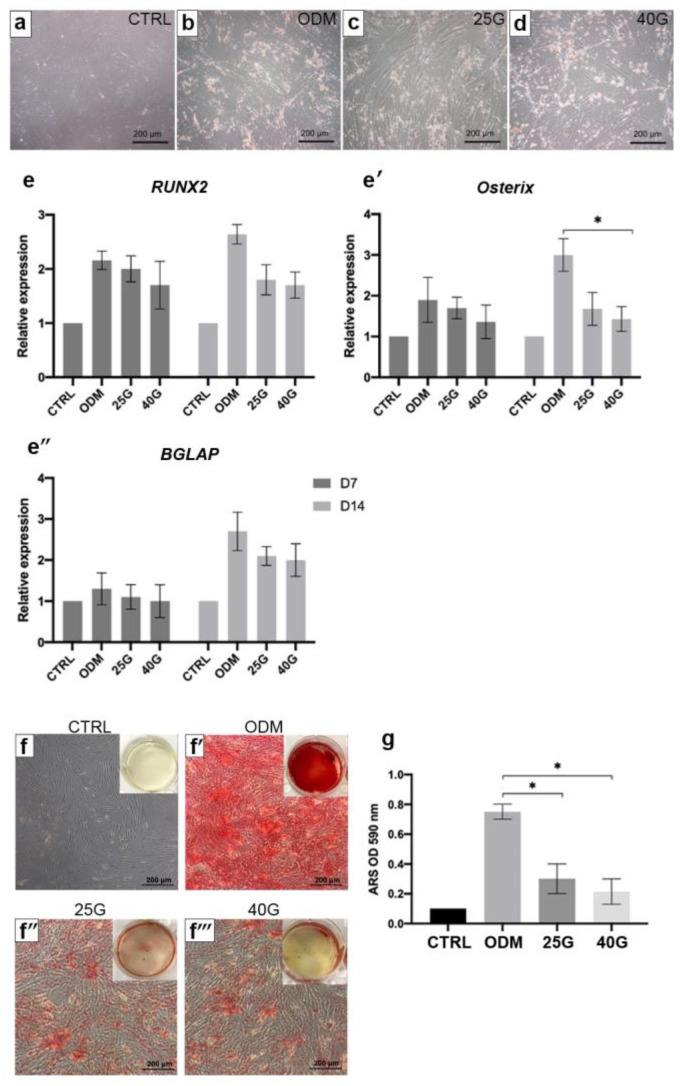
The effect of high levels of glucose on the in vitro osteogenic differentiation of MSCs. The MSCs were cultured in growth medium (control, CTRL), ODM (5.5 mM glucose), ODM with 25 mM glucose (25G), and ODM with 40 mM glucose (40G). Morphology of MSCs in each condition was observed (**a**–**d**). The expressions of RUNX2 (**e**), Osterix (**e’**), and BGLAP (**e’’**) were investigated at day 7 and 14. Gene expression is presented as relative to the CTRL. Alizarin Red S staining at day 14 is shown (**f**–**f’’’**). Semi-quantitative analysis of ARS staining was performed by dye extraction (**g**). All data are presented as the means ± SEM from three independent experiments. * *p* value < 0.05 is considered statistically significant.

The MSCs were cultured in growth medium (5.5 mM glucose, control), ODM (5.5 mM glucose), and ODM with high-glucose concentrations (25 mM and 40 mM glucose) for 21 days. The cell morphology and density in the ODM with 25 mM and 40 mM glucose were similar to those that were in the ODM (Figure 2a–d). The expression patterns of the osteogenic genes, RUNX2, Osterix, and BGLAP were evaluated on days 7 and 14 using the reverse transcription quantitative polymerase chain reaction (RT–qPCR). The transcriptional level of RUNX2, Osterix, and BGLAP (Figure 2e–e’’) were reduced in the high-glucose conditions when it was compared to that of the ODM (5.5 mM glucose). The remarkable reduction in the genes was observed in day 14. Furthermore, the adverse effect of high levels of glucose on the differentiation potential of the MSCs was demonstrated by a matrix mineralization that was stained with ARS at day 21. The accumulation of ARS in the 25 mM and 40 mM glucose conditions was less than those in the ODM ones (5.5 mM glucose) as examined by a macroscopic and microscopic examination (Figure 2f–f’’’). The semi-quantitative analysis of the ARS staining revealed a significant reduction in the ARS staining in the high-glucose conditions when it was compared with that of the ODM (5.5 mM glucose) (Figure 2g). However, the dose-dependent effect of glucose on the osteogenic differentiation of the MSCs was not observed in these experiments.

### 2.3. Alteration of ANXA2 Expression in MSC Osteoblastic Differentiation under High-Glucose Conditions

**Figure 3:**
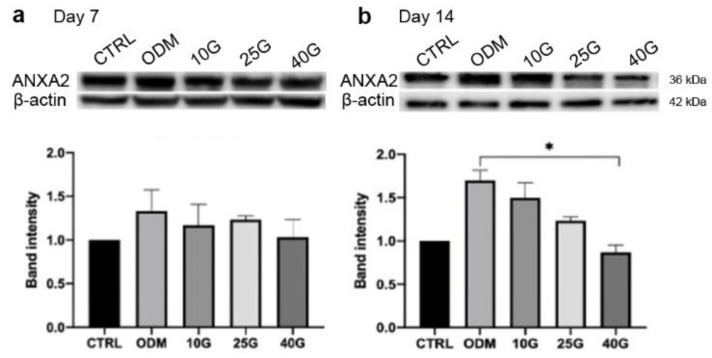
The expression of ANXA2 in MSCs during osteogenic differentiation under high-glucose condition. Western blot analysis demonstrated ANXA2 expression at day 7 (**a**) and day 14 (**b**) in MSCs in growth medium (CTRL), ODM (5.5 mM glucose), ODM with 10 mM (10G), 25 mM (25G), and 40 mM (40G) glucose. The protein band intensity is presented as relative to CTRL. All data are presented as the means ± SEM from three independent experiments. * *p* value < 0.05 is considered statistically significant.

The expression of the intracellular ANXA2 was determined by a Western blot analysis after the in vitro MSC osteoblastic differentiation in the presence of 10 mM, 25 mM, and 40 mM glucose for 14 days. The expression of ANXA2 at day 7 revealed that there was no difference among the conditions (Figure 3a). At day 14, the high levels of glucose gradually decreased the expression of ANXA2 in the MSC osteoblastic differentiation in a dose-dependent manner (Figure 3b). A significant reduction in ANXA2 was observed in the 40 mM glucose condition when it was compared with that of the ODM one (5.5 mM glucose). Taken together, these data indicated the impact of high levels of glucose on the expression of ANXA2 in the MSCs during osteogenesis, which might be the cause of the deteriorated osteogenic differentiation potential of MSCs.

### 2.4. The Improvement of In Vitro Osteogenic Differentiation of MSCs by Recombinant Annexin A2 (rANXA2)

**Figure 4:**
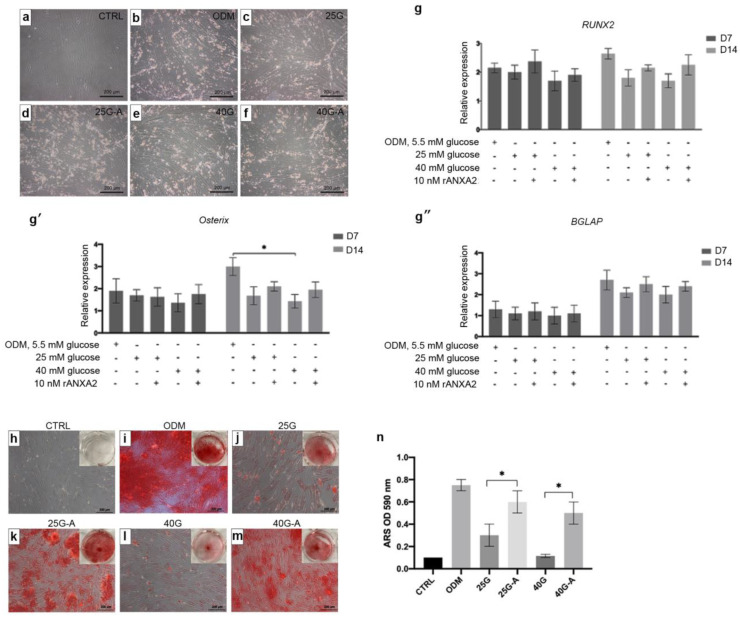
Effect of annexin A2 on MSC osteoblastic differentiation under high-glucose conditions. The recombinant annexin A2 (rANXA2) was added to the ODM with 25 mM glucose (25G-A) and ODM with 40 mM glucose (40G-A) condition. The morphology of MSCs in growth medium (control, CTRL) and in ODM under normal and high-glucose conditions with/without rANXA2 was observed (**a**–**f**). The transcriptional levels of RUNX2 (**g**), Osterix (**g’**), and BGLAP (**g’’**) at day 7 and 14 were determined. Gene expression is presented as relative expression to CTRL. At day 14, matrix mineralization by ARS staining was evaluated (**h**–**m**). The accumulation of ARS was extracted and measured at optical density 590 nm (**n**). Data are presented as the means ± SEM from three independent experiments. * *p* value < 0.05 is considered statistically significant.

To demonstrate whether rANXA2 could recover the deteriorated osteogenic differentiation of the MSCs that were affected by the high levels of glucose, 10 nM rANXA2 was added to the high-glucose conditions, which were the 25 mM and 40 mM glucose ones. At day 14, the micrograph revealed that there were no morphological differences among the conditions (Figure 4a–f), whereas the transcriptional levels of RUNX2, Osterix, and BGLAP showed that there was a slight increase in them when rANX2 was added to the high-glucose conditions (Figure 4g–g’’). The effect of rANXA2 on the matrix mineralization of the MSCs was evaluated by an ARS staining at day 21. The treatment with rANXA2 significantly increased the ARS accumulation of the MSCs that were differentiated under high-glucose conditions (Figure 4h–n). Collectively, the deteriorated osteogenic differentiation of the MSCs under the high-glucose conditions might be governed by the alteration of the ANXA2 expression, which could be recovered by a treatment with rANXA2.

### 2.5. rANXA2 Attenuated High Glucose-Induced Cellular Senescence of MSCs during Osteogenic Differentiation

**Figure 5:**
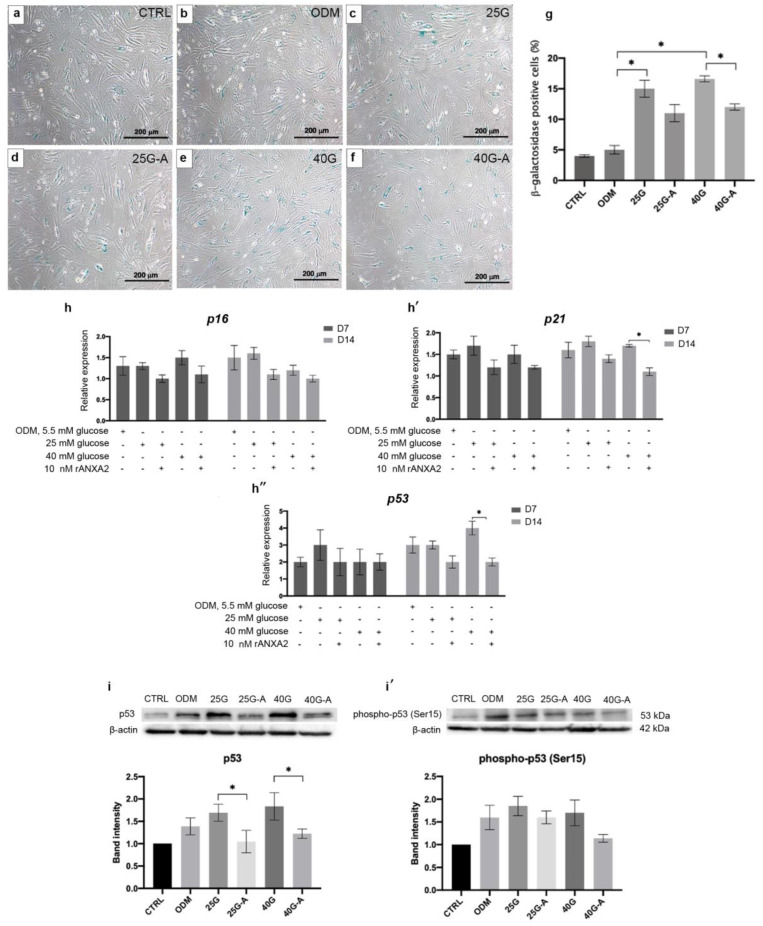
The impact of annexin A2 on cellular senescence of MSCs cultured in ODM with high levels of glucose. MSCs were cultured in ODM under normal (5.5 mM glucose) and high-glucose condition with/without rANXA2 (25G vs. 25G-A, 40G vs. 40G-A). Micrograph show the SA-β-gal positive cells which stained blue in each conditions (**a**–**f**). Total cells and SA-β-gal positive cells were counted and calculated (**g**). The transcriptional levels of p16, p21, and p53 were determined at day 7 and 14 (**h**–**h’’**). Gene expression is presented as relative to CTRL. Western blot analysis of p53 (**i**) and phosphorylated p53 (**i’**) was determined at day 14. Protein band intensity is present as relative to CTRL. All data are presented as the means ± SEM from three independent experiments. * *p* value < 0.05 is considered statistically significant.

It has been demonstrated that a high-glucose concentration induces the cellular senescence of the MSCs. To examine whether ANXA2 was associated with this consequence, the senescence-associated beta-galactosidase (SA-β-gal) was examined in the MSCs during the osteoinduction under the high-glucose conditions. After 7 days of osteoinduction, the senescent cells were significantly increased in the high-glucose conditions when they were compared with that in ODM (5.5 mM glucose). The treatment with rANXA2 could significantly reduce the senescent cells in the high-glucose conditions (Figure 5a–g). The transcriptional levels of the essential senescence-associated genes were determined. There was no significant difference in the p16, p21, and p53 expression between the control ODM (5.5 mM glucose) and the high-glucose ODM (25 mM and 40 mM glucose). However, the addition of rANXA2 to the high-glucose conditions downregulated the expression of p16, p21, and p53. The marked reduction in the genes was observed at day 14 in the 40 mM glucose condition (Figure 5h–h’’). A Western blot analysis revealed that there was a significant reduction in p53 in the presence of rANXA2 in the ODM with the high-glucose conditions in both the 25 mM and 40 mM glucose tests (Figure 5i). The expression of phosphorylated p53 (Ser15), which is the active form, was also reduced by the rANXA2 treatment, however, it was not significantly different when it was compared with that in the high-glucose conditions (Figure 5i’). Taken together, these results suggest that rANXA2 may improve in vitro osteogenic differentiation of the MSCs under the high-glucose conditions by reducing the cellular senescence.

## 3. Discussion

Long-term exposure to high blood glucose in the human body could lead to the dysfunction of several organs due to oxidative stress and chronic inflammation occurring. Organ dysfunction that is influenced by hyperglycemia is frequently found in many organs, such as the cardiovascular system, the eyes, the nervous systems, and the bones. Moreover, chronic complications such as secondary osteoporosis are commonly found in patients with long-term exposure to high blood sugar levels, thereby leading to bone tissue disturbances. The exact mechanism of how hyperglycemia leads to low bone mass is particularly important for the diagnosis, prognosis, and treatment of it; however, it is still largely unknown. Recently, great attention has been given to the osteoblasts, which are bone-forming cells that are influenced by high blood glucose during bone formation. In the present study, the in vitro MSC osteoblastic differentiation in different glucose concentrations was performed. To reflect the normal and high blood glucose levels in humans, 5.5 mM, 10 mM, 25 mM, and 40 mM glucose, which are approximately equal to 100, 180, 450, and 720 mg/dl, respectively, were employed. The impairment of the osteogenic differentiation of the MSCs under the high-glucose conditions was demonstrated by the reduction in the osteogenic gene expression and matrix mineralization. The results were concordant with the findings from other studies which investigated the impact of high-glucose levels on MSC proliferation and differentiation. Wang J et al. [20] found that an inhibitory effect of high levels of glucose (25 mM) on the alkaline phosphatase activity and the matrix mineralization of the MSCs through the suppression of bone morphogenetic protein 2 (BMP) signaling. Moreover, a high level of glucose-induced oxidative stress consequently reduced the growth rate and increased the apoptotic gene expression in gingival MSCs [21]. A high-glucose microenvironment and oxidative stress have been found to be associated with MSC senescence [9,22]. As demonstrated in the present study, the SA-β-gal-positive cells were increased in the MSCs during the osteogenic differentiation with high-glucose ODM. These suggest that triggering senescence might be the plausible cause that is underlined in the glucose-induced deterioration of the osteogenic differentiation potential of the MSCs.

In recent years, the alteration of many proteins and their roles in bone development have received attention in the exploration of the pathogenesis of secondary osteoporosis in diabetic patients. Annexin A2, a member of the calcium-binding protein family, plays diverse roles in many conditions, such as inflammation [23] and matrix mineralization [24], and particularly in bone formation [17]. The proteomic study of the biomarkers in type 2 DM patients revealed that ANXA2 was down-regulated in the patients when they were compared with the healthy controls [25]. The reduction in ANXA2 has been reported to be associated with an increased glucose concentration in the endothelial cell culture in vitro [26]. Our study demonstrated an alteration of the ANXA2 expression in the MSCs during in vitro MSC osteoblastic differentiation under high-glucose condition. The defective osteogenic differentiation as a result of a high-glucose concentration was improved by adding rANXA2 to the culture system. In particular, the matrix precipitation was markedly improved as examined via ARS staining. These findings were related to the study of the human osteosarcoma cells, showing the role of ANXA2 in the terminal stage of osteogenic differentiation, particularly in matrix mineralization [27]. Additionally, ANXA2 has been identified as an essential protein in the extracellular vesicles that are secreted from mineralizing osteoblasts to promote the mineralization of human MSCs [28]. Interestingly, the addition of rANXA2 to the high-glucose conditions, the downregulation of the senescence-associated genes: p21 and p53, and reduction in the SA-β-gal-positive cells were observed. A Western blot analysis revealed the significant reduction in the total p53 protein after employing rANXA2; however, the serine 15-phosphorylated form of p53 was not significantly reduced. This result can be explained by the study by Ashcroft M et al., showing the stabilization of p53 in response to DNA damage without any phosphorylation occurring [29]. Hyperglycemia induces cellular damage via triggering several stimuli including the reactive oxygen species (ROS) and oxidative stress [9,21,22]. The elevated ROS causes direct DNA damage and leads to the activation of the p53 pathway, which correlated with the induction of either senescence or the apoptosis of the cells [30]. P53 plays a vital role in initiating cellular senescence by activation of the p53/p21 pathway, thereby resulting in cell cycle arrest [31]. In the present study, the addition of ANXA2 could reduce the expression of p53 and p21 that are induced by the high-glucose conditions, thus suggesting that ANXA2 attenuated the high glucose-induced cellular senescence by suppressing the p53 pathway. The association of ANXA2 and p53 has been investigated in cancers. The overexpression of ANXA2 in cancer cells that is correlated with cancer cell proliferation and knockdown of ANXA2 was found to increase the expression of p53, which led to cell cycle arrest [32,33]. Moreover, ANXA2 could regulate the ROS production in the inflammatory response to polymicrobial sepsis [34].

Taken together, high-glucose conditions influenced ANXA2, which may induce the cellular senescence of the MSCs through the activation of the p53/p21 pathway, but the addition of rANXA2 recovered this outcome. Cellular senescence was demonstrated to influence the bone formation in many studies [35,36]. However, the expression of ANXA2 can be regulated through various substances, including those that are involved with inflammation and cancers [37,38].

## 4. Material and Methods

### 4.1. MSC Isolation and Expansion

Fresh human bone marrow was aspirated from three healthy donors. Samples were collected under aseptic conditions after subjects provided written informed consent. The ethical approval of this study was granted by the Ethical Committee of Faculty of Medicine, Ramathibodi Hospital, Mahidol University (COA.MURA2019/893). The protocol of cell isolation is briefly described as follows: aspirated bone marrow was diluted with red blood cell lysis buffer at a 1:5 ratio and incubated at room temperature for 10 min. Samples were centrifuged at 400 g at 25 °C for 10 min. The cell pellet was collected, seeded into 6-well plates, and cultured with growth medium containing Dulbecco’s Modified Eagle Medium-low glucose (DMEM, Gibco, Grand Island, NY, USA), 10% fetal bovine serum (FBS, Merck Millipore, Burlington, MA, USA), 100 U/mL penicillin, and 100 µg/mL streptomycin (Gibco, Grand Island, NY, USA). The cells were maintained at 37 °C in a humidified atmosphere containing 5% CO_2_. After 48 h, the non-adherent cells were removed. The medium was changed every 3 days. Sub-culture was performed when the cells reached 80% confluence using 0.25% Trypsin-EDTA. MSCs at passages 4th−6th were employed in this study.

### 4.2. MSC Characterization

According to the minimal criteria of the International Society for Cellular Therapy (ISCT), all of the MSC samples were characterized using cell surface markers and multilineage differentiation potential.

### 4.3. Cell Surface Markers

At the indicated passages, MSCs were trypsinized and washed twice with phosphate-buffered saline (PBS). MSCs were adjusted to 100,000 cells per tube, and this step was followed by staining them with mouse monoclonal antibodies as follows: CD34-PE (BD Biosciences, Franklin Lakes, NJ, USA), CD45-PerCP (BD Biosciences, Franklin Lakes, NJ, USA), CD73-PE/Cy7 (BD Biosciences, Franklin Lakes, NJ, USA), CD90-APC (BD Biosciences, Franklin Lakes, NJ, USA), and CD105-PE (BD Biosciences, Franklin Lakes, NJ, USA). Cells were incubated at 4 °C for 30 min. Then, the cells were washed twice with PBS and fixed with 1% paraformaldehyde. Stained cells were examined using a FACSCanto^TM^ II flow cytometer (BD Biosciences, Franklin Lakes, NJ, USA). Data were analyzed using FACSDiva software version 6.1.3 (BD Biosciences, Franklin Lakes, NJ, USA).

### 4.4. Osteogenic, Adipogenic, and Chondrogenic Differentiation

MSCs were trypsinized, washed twice with PBS, and seeded into 6-well plates at a density of 1 × 10^5^ cells per well. After 24 h of culture, the medium was changed to osteogenic differentiation medium (ODM) containing growth medium, 0.1 μM dexamethasone, 10 mM β-glycerophosphate, and 50 μg/mL ascorbic acid (all from Sigma–Aldrich, St. Louis, MO, USA). After 2 weeks, Alizarin Red S (ARS) staining was used to examine matrix mineralization. For semi-quantitative analysis of ARS staining, ARS-stained cell monolayer was eluted with 10% acetic acid for 30 min at room temperature with gentle shaking. The eluate was collected and heated at 85 °C for 10 min. After centrifugation at 900× *g* for 10 min, the supernatant (100 µL) was collected and mixed with 100 µL of 10% ammonium hydroxide before the absorbance was measured at 405 nm.

For adipogenic differentiation, the MSCs were cultured in adipogenic differentiation medium (ADM) containing growth medium, 0.5 mM 3-isobutyl-1-methylxanthine (IBMX), 1 µM dexamethasone, 10 µM insulin, and 200 µM indomethacin (all of them were purchased from Sigma–Aldrich, St. Louis, MO, USA). After 2 weeks, Oil Red O staining was used to determine the characteristics of adipocyte-like cells.

For chondrogenic differentiation, the MSCs were cultured in StemPro™ chondrogenic differentiation medium (CDM, Gibco, Grand Island, NY, USA) for 10 days before staining with Alcian blue to determine the expression of glycosaminoglycans.

### 4.5. The Effects of High Glucose on MSC Osteoblastic Differentiation

To determine the impact of high levels of glucose on in vitro osteoinduction, MSCs at the fourth passage (*n* = 3) were seeded into 6-well plates at a density of 1 × 10^5^ cells per well. MSCs were cultured in ODM that was supplemented with various concentrations of glucose (Sigma Aldrich, St. Louis, MO, USA) as follows: 10 mM (10G), 25 mM (25G), and 40 mM (40G) glucose for 14 days. For control, MSCs were cultured in standard ODM which contained 5.5 mM glucose. Cell morphology, osteogenic gene expression, matrix mineralization by ARS staining, and senescence assay were examined. The effect of annexin A2 was examined by using 10 nM human recombinant annexin A2 (rANXA2, Abcam, ab93005, Boston, MA, USA).

### 4.6. Reverse Transcription Quantitative Polymerase Chain Reaction (RT-qPCR)

Cells were harvested in TRIzol (Thermo Fisher Scientific, Waltham, MA, USA). Total RNA purification was performed using Direct-zol RNA Mini Prep (Zymo Research, Irvine, CA, USA) following the manufacturer’s protocols. RNA concentration and purity were determined using a Thermo Scientific Nanodrop^TM^ 2000 spectrophotometer. Then, cDNA was synthesized from 1 μg of total RNA using iScript^TM^ Reverse Transcription Supermix (Bio-Rad, Hercules, CA, USA) according to the manufacturer’s instructions. The expression levels of the genes of interest (Table 1) were determined using the SYBR Green method and normalized to the endogenous housekeeping gene, glyceraldehyde-3-phosphate dehydrogenase (GAPDH). Real-time PCR was performed using a CFX96^TM^ Real-Time PCR Detection System (Bio-Rad, Hercules, CA, USA). Data are presented as a relative expression compared to the control sample.

### 4.7. Western Blot Analysis

To perform protein analysis, the whole cell lysate of MSCs was harvested on ice using cold RIPA lysis buffer (Merck Millipore) containing protease inhibitor (Thermo Fisher Scientific) and phosSTOP^TM^ (Sigma–Aldrich, St. Louis, MO, USA). A total of 30 µg protein was loaded on a 12% sodium dodecyl sulfate–polyacrylamide gel electrophoresis (SDS–PAGE) gel and transferred to a polyvinylidene fluoride (PVDF) membrane. The membrane was submerged in the primary antibody at 4 °C overnight, and this step was followed by the addition of a secondary antibody at room temperature for 2 h. The list of antibodies that were used are as follows: rabbit anti-Annexin A2 (1:10,000 diluted; Abcam, Boston, MA, USA), rabbit anti-phospho-p53 (1:1000 diluted; Cell Signaling Technology, Danvers, MA, USA), mouse anti-p53 (1:1000 diluted; Cell Signaling Technology, Danvers, MA, USA), and mouse anti-ß-actin (1:20,000 diluted; Merck Millipore, Burlington, MA, USA), HRP-labeled goat anti-rabbit IgG (1:10,000 diluted; Cell Signaling Technology, Danvers, MA, USA), and HRP-labeled goat anti-mouse IgG (1:10,000 diluted; Abcam, Boston, MA, USA). The protein bands were visualized using enhanced chemiluminescence (ECL, Amersham ECL Prime Western Blotting Detection Reagent, GE Healthcare, Chicago, IL, USA) and chemiluminescence detection using a ChemiDoc^TM^ MP Imaging System (Bio-Rad, Herakles, CA, USA). The band intensity was quantified and normalized to that of β-actin using Image Lab^TM^ software Version 5.2.1 (Bio-Rad, Hercules, CA, USA).

### 4.8. Senescence-Associated Beta-Galactosidase (SA-β-Gal) Staining

MSCs from the fourth passage (*n* = 3) were seeded into 6-well plates at a density of 5 × 10^4^ cells per well and cultured in growth medium and ODM with various concentrations of glucose for 7 days. Cells were examined for SA-β-gal activity according to the manufacturer’s protocol (Cell Signaling Technology, Danvers, MA, USA). The senescent cells which stained blue were observed using inverted microscope. Number of SA-β-gal-positive cells were counted and presented as percentage to total cell (1000 cells).

### 4.9. Statistical Analysis

All of the data were analyzed, and they are presented as the means ± standard error of the mean (SEM) from three individual experiments. Statistical analysis was performed using PASW Statistics, version 18. One-way analysis of variance (one-way ANOVA) and post hoc analysis using Tukey’s multiple comparisons was used to compare differences among groups. A *p* value less than 0.05 was considered statistically significant.

## 5. Conclusions

A hyperglycemic condition can lead to many organ dysfunctions including those of the bone. Osteogenesis is an orchestrated process involving many cell types. Our study demonstrated that the osteo-induction disruption under high-glucose conditions may be partly influenced by the alternated expression of ANXA2. The reduction in the senescent rate might be one of the plausible mechanisms for ANXA2 supporting the osteogenic differentiation in a diabetic condition. Hence, our results were a piece of crucial data demonstrating the alleged factor’s involvement in osteoblastogenesis in high levels of glucose. However, exploring in-depth mechanisms on bone fractures in diabetic patients is highly needed to develop and improve the therapeutic approaches, particularly for the biomarkers of it, in the future.

## Figures and Tables

**Table 1:** Primer sequence for detecting mRNA expression.

	Target Gene	Nucleotide Sequence	Product Size (bp)
Forward	RUNX2	5′-AACCGAGAAGGCACAGACAG-3′	192
Reverse	RUNX2	5′-GCCTGGGGTCTGTTATCTGA-3′	
Forward	Osterix	5′-TGCTTGAGGACGAAGTTCAC-3′	114
Reverse	Osterix	5′-GTGCTTTGCCCAGAGTTGTT-3′	
Forward	BGLAP	5′-TTTGTCCAAACCATCCGCAC-3′	154
Reverse	BGLAP	5′-GCATCAACTTCGATACCGGC-3′	
Forward	p16	5′-TGAGGGTTTTCGTGGTTCAC-3′	190
Reverse	p16	5′-TGGTCTTCTAGGAAGCGGC-3′	
Forward	p21	5′-GATGAGTTGGGAGGAGGCAG-3′	210
Reverse	p21	5′-CTGAGAGTCTCCAGGTCCAC -3′	
Forward	p53	5′-ATGATTTGATGCTGTCCCCG-3′	175
Reverse	p53	5′-CAAGAAGCCCAGACGGAAAC-3′	
Forward	GAPDH	5′-CAACTACATGGTTTACATGTTCGAA-3′	206
Reverse	GAPDH	5′-CAGCCTTCTCCATGCTGGT-3′	

RUNX2, Runt-related transcription factor 2; BGLAP, bone gamma-carboxyglutamic acid-containing protein; p16, cyclin-dependent kinase inhibitor 2A; p21, cyclin-dependent kinase inhibitor 1A, p53, Tumor protein P53; GAPDH, Glyceraldehyde 3-phosphate dehydrogenase.

## Data Availability

All the data analyzed in this study are included in this published article.
